# Exploring the Influence of a Diabetes Specialty Outpatient Clinic on Adolescents With Type 1 Diabetes in Barbados: A Qualitative Study

**DOI:** 10.1155/pedi/5454172

**Published:** 2025-12-03

**Authors:** Gemma-Ann Benskin, Paula Michele Lashley

**Affiliations:** Department of Clinical Sciences, University of the West Indies, Cave Hill Campus, Bridgetown, Barbados

## Abstract

**Objectives:**

This study explored the perceptions of adolescents with type 1 diabetes mellitus (T1DM) regarding their self-management and the impact of a diabetes specialty outpatient clinic on their quality of life (QOL) in Barbados.

**Design:**

A qualitative, descriptive–interpretive study using semi-structured online interviews.

**Setting:**

Paediatric diabetes specialty outpatient clinic at the Queen Elizabeth Hospital (QEH), Barbados.

**Participants:**

Twelve adolescents aged 13–17 years with T1DM for > 1 year who attended the diabetes specialty outpatient clinic for at least 6 months.

**Methods:**

Interviews were transcribed verbatim, coded using ATLAS.ti 23, and analysed thematically using a constant comparison approach.

**Results:**

Three organising themes—autonomy, internal resilience and clinic and social support—contributed to the global theme of diabetic health literacy. Participants demonstrated varied levels of diabetes self-management confidence. Clinic interactions, family support and peer understanding were key influences on autonomy and resilience. Adolescents identified a need for age-appropriate communication and psychosocial support.

**Conclusions:**

Diabetic health literacy among Barbadian adolescents is influenced by clinical support, psychosocial resources, and educational strategies. Adolescents' autonomy should be fostered through youth-centred approaches that enhance self-efficacy and support transition readiness.

## 1. Introduction

Type 1 diabetes mellitus (T1DM) is the third most prevalent chronic disease in childhood and the most frequent type of diabetes in children [1]. The incidence of T1DM in the paediatric population has been increasing worldwide since 1960 at an approximate 3%–5% yearly rate [2].

In particular, adolescence is a very challenging period in their lives as these children are developing their sense of autonomy and beginning to broaden their focus from salient tasks of childhood to include emerging tasks of adulthood [3]. Adolescents with T1DM cope with the usual physical and psychosocial burden as their peers whilst also simultaneously managing the added stressor of a chronic illness. Furthermore, puberty occurs during this period, and the rapid growth and hormonal changes are often characterised by deterioration in glycaemic control [4]. Adherence to and compliance with treatment regimens become difficult concepts for many adolescents living with T1DM to grasp, leading to further poor glycaemic control and increasing the risk of developing serious health problems and reduces life expectancy [5, 6].

Adolescents experience a perpetual, daily struggle of having to conform to a prescriptive way of life relating to the disease process, emotions, their social world, the practical aspects of having diabetes and health care maintenance. This is likely compounded by poor communication between patients and healthcare professionals in the adolescent T1DM population [7]. Qualitative research has shown that adolescents experience extra effort, restriction, pain, and additional worries because of chronic illness [8, 9]. These issues are increasingly being recognised by health professionals and also by the adolescents themselves. Studies have shown that their needs are often not adequately met or may even be ignored in adolescents with chronic conditions [9–11]. Moreover, it has been shown that a comprehensive approach improves the outcome of the chronic condition itself [12].

The main aims of diabetes care in paediatric patients are to achieve optimal glycaemic control, normal psychosocial development, and support for the young person and family in developing strategies to cope with a lifetime of diabetes. A major challenge is maximising the quality of life (QOL) of the adolescent in the context of effective therapeutic interventions [13]. Adolescents with T1DM who reported better QOL had lower levels of glycohemoglobin, fewer acute events and hospitalisations [14]. A multidisciplinary team of specialists trained in paediatric diabetes management is necessary, a team who is sensitive to the challenges that adolescents with type 1 diabetes and their families face. It is essential that diabetes self-management education and support, medical nutrition therapy, and psychosocial support be provided at diagnosis and regularly thereafter [15]. In Barbados, this is primarily done in the diabetes specialty outpatient clinic where physicians and diabetes educator nurses form the basis of the team ensuring that all concerns are addressed and compliance with other necessary appointments. Thus, one major way to impact QOL is likely through well-designed outpatient clinic care and patient education.

Adolescence is the point of transition to adulthood and appropriate education within outpatient clinics could result in long-term optimal control and good diabetes management. In turn, good diabetes management in adolescence is associated with decreased adult admissions and diabetes complications in the future [16]. Thus far, we know T1DM is a prevalent childhood disease worldwide, but we know little about the influence of outpatient clinics on adolescents in this population relating to their diabetes control and management.

Outpatient education is less expensive than inpatient education and is also associated with better diabetes outcomes when there is availability of appropriate resources [17]. Continued education on diagnosis and regularly as an outpatient is the key to well-controlled diabetes and improved QOL. Diabetes specialty outpatient clinics with specialised nurses are the cornerstones to achieving this goal and decreasing concerns regarding frequent hospitalisations, poor school performance and microvascular and macrovascular complications in the paediatric population [18]. Twenty years ago, Delamater made a call for the development and refinement of interventions to improve QOL in children with diabetes [19]. Though internationally some progress has been made, no explicit attempts have been documented in the Caribbean.

In the Caribbean, diabetes care is influenced by distinctive sociocultural and health system characteristics. Barbados, as a small island developing state (SIDS), relies on a single tertiary referral hospital, where shortages of diabetes educators, clinic staff and resource constraints shape outpatient care. Adolescents' experiences are further influenced by strong family involvement in medical decision-making, cultural attitudes towards chronic illness and stigma in educational settings [20, 21]. These contextual elements highlight the originality of this study, as no previous qualitative work has explicitly examined how adolescents perceive their clinic experience and the impact on diabetic health literacy and QOL in the Caribbean.

## 2. Methods

### 2.1. Study Design and Patient Involvement

This was a qualitative study using descriptive–interpretive methodology and utilised semi-structured interviews using a secure online platform—Zoom Video Communications Inc. (Zoom). Adolescents with type 1 diabetes were involved in shaping the research focus and interview content. The research question and semi-structured interview schedule were informed by clinical observations of adolescent patients with T1DM attending the diabetes specialty outpatient clinic and informal feedback gathered during routine care. Adolescents aged 13–17 years were purposively sampled and provided input on their experiences, concerns, and suggestions regarding diabetes care, clinic interaction and QOL. The findings directly reflect their lived experiences and perspectives and are intended to inform future service improvements to outpatient care.

### 2.2. Setting

The study was conducted in the paediatric diabetes specialty outpatient clinic at the Queen Elizabeth Hospital (QEH), the sole tertiary public hospital in the SIDS of Barbados.

### 2.3. Participants

Purposive sampling with maximum variation was performed as guided by the eligibility criteria. Our participant selection included adolescents (aged 13–17 years) with a diagnosis of T1DM for > 1 year and enrolled in the paediatric diabetes specialty outpatient clinic at the QEH for at least 6 months regardless of treatment type with the exclusion of acutely ill hospitalised T1DM adolescents or those known to be under active management for a significant psychiatric illness.

### 2.4. Data Collection

Data were collected from January to March 2023 using online semi-structured interviews by a trained research assistant. Interviews averaged 15 min and were transcribed verbatim 50:50 by a qualitative transcription professional and the principal investigator (PI). Data saturation was reached when no new codes or themes emerged after the 10^th^ interview with subsequent transcripts confirming thematic consistency [22]. All transcripts were de-identified and coded and stored securely in a password protected folder on an encrypted computer.

### 2.5. Analysis

Interview content was reviewed, and an initial 16-item inductive coding dictionary was developed (Table 1) with inter-rater reliability checks. We conducted thematic analysis on each transcript using ATLAS.ti23; basic themes were identified by coding frequencies and clusters and further condensed to three organising themes and then one global theme. Data saturation was reached when no new codes or themes emerged after the 10^th^ interview with subsequent transcripts confirming thematic consistency [22]. Reflexivity was addressed by ongoing reflection on how the investigators' professional background in paediatrics and diabetes care might influence rapport and interpretation [23]. To enhance rigour, coding was cross-checked between the PI-(GB) and NG (a doctoral level trained qualitative supervisor) and an audit trail of coding decisions was maintained. Although member checking was not conducted directly with participants, findings were shared with the broader clinical team for feedback contributing to credibility and validity.

## 3. Results

### 3.1. Participant Characteristics

Twelve of the 21 eligible participants were interviewed. Reasons for non-participation included migration, limited access to reliable internet connection and unwillingness. Participants ranged in age from 13 to 16 years and duration of living with T1DM spanned from 3 to 12 years (Table 2).

### 3.2. Thematic Findings

Thematic analysis generated three organising themes—autonomy, internal resilience, and clinic and social support—which collectively informed the global theme of diabetic health literacy. This global theme reflects how adolescents in Barbados engage with health services, family and peer networks to manage their diabetes and enhance the QOL.

#### 3.2.1. Autonomy

Participants demonstrated varying levels of self-management and decision-making, influenced by their understanding of diabetes, emotional readiness and support structures. Some expressed confidence in taking charge of their diabetes care:

“I put more effort into it now I'm older… I have not gone back to hospital because of diabetes since I was diagnosed.” (P01)

“I know I can take care of my diabetes myself and be well balanced.” (P09)

Others acknowledged dependence on parental interpretation of clinic information:

“If I don't understand it, my father most likely going understand it, so when he finish, he explain it to me.”(P03)

“My mum is usually the one trying to make sure that my blood sugars are in check.” (P11)

Some adolescents highlighted that complex medical explanations from clinicians limited their ability to act independently:

“The information was more towards the adults… it needed to be simpler and broken down for my age.” (P02)

“They have not directly showed (me how to manage) in a way that I would understand mostly.” (P07)

Mood and motivation also played a role:

“Sometimes I would be excited… but if I'm in that mood of I just don't care about diabetes, it doesn't really change.” (P02)

#### 3.2.2. Internal Resilience

Participants described personal coping strategies to adapt to the challenges of living with diabetes. Some demonstrated emotional growth and acceptance:

“I never let it [diabetes] stop me from doing anything I wanted to do.” (P05)

“I feel a lot better… I still allowed myself to enjoy life.” (P09)

Others expressed ongoing frustration, particularly with injections, food restrictions, and social situations:

“It's very tiring… I know I have to do it because if I don't I'll get sick.” (P08)

“I feel like if I was robbed of all these things… I don't really want to go out so with friends. I feel like I'm just odd.” (P02)

Some adolescents expressed a sense of emotional fatigue or feeling misunderstood:

“Sometimes I just don't feel like doing it, but I know I have to… it's very tiring.” (P05)

Despite these challenges, several participants described actively trying to stay positive or seeking help:

“My friends know about my diabetes… sometimes I get mad but I know they are trying to help.” (P03)

“I talk to a friend and they tell me don't give up. Keep trying… gives me confidence.” (P12)

#### 3.2.3. Clinic and Social Support

Experiences with the paediatric diabetes outpatient clinic were mixed. Many adolescents reported that clinic staff were supportive and provided useful education:

“I like coming to the clinic… the nurses and doctors encourage me and respond to my worries.” (P12)

“They teach me how to take care of my diabetes in a way that fits me.” (P11)

However, some felt the focus was too medical or lacked emotional support:

“It's not just insulin and blood sugars… there should be more care and training to understand diabetes.” (P04)

“Nobody ever tries to make it differently so you can understand it better or more fun.” (P07)

Peer support from other adolescents with diabetes was particularly valued:

“If there is a problem… we help and support each other, and we get through it better.” (P05)

Several participants shared difficult experiences at school, including exclusion from activities or misunderstanding from teachers:

“They don't let me do nothing since I develop this… I used to run for the school.” (P08)

“They stopped me from doing sports… thought I wasn't capable because of my diabetes.” (P07)

Family and close friends were identified as key sources of practical and emotional support:

“My friends help me do my blood sugar or show the teachers in case something goes wrong.” (P07)

“Everybody in my family knows… they help me tread lightly with it.” (P12)

### 3.3. Global Theme: Diabetic Health Literacy

**Figure 1:**
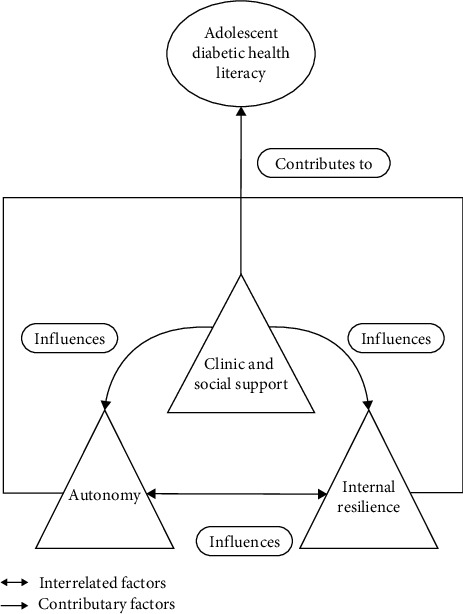
Factors influencing adolescent diabetic health literacy. Key ↔ Interrelated factors; → Contributary factors.

The interplay of autonomy, resilience, and support culminated in the global theme of diabetic health literacy—the ability of adolescents to access, understand, and use diabetes-related information in their daily lives. This theme encapsulates their readiness to engage with medical advice, adapt lifestyle behaviours, and make informed decisions for long-term diabetes self-management and well-being.  Figure 1 illustrates the interrelated clinical, psychosocial and personal factors shaping adolescent diabetic health literacy.

## 4. Discussion

This study explored the lived experiences of adolescents with T1DM in Barbados and how they perceive the influence of a diabetes specialty outpatient clinic on their self-management and QOL. Through thematic analysis of in-depth interviews, we identified three key organising themes—autonomy, internal resilience and clinic and social support—which together inform the global theme of diabetic health literacy. These findings highlight how adolescents interact with health services and social environments to navigate their chronic condition, particularly within a SIDS context.

### 4.1. Autonomy in Adolescent Diabetes Care

Autonomy emerged as a dynamic process shaped by age, emotional readiness, and the accessibility of health information. Adolescents who had lived longer with their diagnosis expressed greater confidence in self-management, echoing previous studies that link illness duration and maturity with improved diabetes self-efficacy [13–16]. However, younger or less experienced participants often relied on parental interpretation of clinic instructions, underscoring the need for age-appropriate communication within outpatient settings [6]. When clinical information was perceived as overly technical or adult-centric, adolescents reported disengagement or confusion—highlighting a barrier to effective participation in their own care.

Fostering autonomy in adolescent diabetes care is essential to successful transition to adult services. As recommended by the International Society for Pediatric and Adolescent Diabetes (ISPAD), clinicians should promote gradual independence by engaging adolescents directly in goal-setting, medication planning, and decision-making [24]. This study reinforces that clear, comprehensible dialogue is a foundational tool in building trust and responsibility among youth with T1DM.

### 4.2. Internal Resilience and Emotional Coping

Participants' narratives reflected varying levels of emotional resilience, ranging from optimism and acceptance to frustration and burnout. Several adolescents described making lifestyle adjustments to maintain blood glucose control without compromising social or academic activities. Others, however, expressed fatigue from the burden of daily disease management, aligning with global estimates that 11%–50% of adolescents with T1DM experience diabetic distress [25].

Emotional resilience is critical for long-term glycaemic control, and psychosocial stressors can directly impact clinical outcomes such as HbA1c and frequency of acute events [19–26]. In our study, adolescents described isolation from peers and feelings of being ‘different,' especially in social situations involving food or injections. These findings suggest the need for clinic models that not only educate but also support emotional regulation and mental well-being, particularly during the vulnerable period of adolescence.

The role of family and peer support in enhancing resilience was consistently acknowledged. Adolescents who reported supportive networks—whether through school, friends, or online groups—felt more motivated and capable in their diabetes self-care. These findings support the implementation of structured peer mentoring or group education programmes within outpatient services to foster shared experiences and reduce feelings of isolation.

### 4.3. The Role of Clinic and Social Support

Participants largely valued their clinic experiences, describing them as educational and encouraging. However, several adolescents voiced concerns that the clinic interactions were overly focused on medical compliance, with insufficient attention paid to the psychological aspects of chronic illness. Some participants reported feeling ‘scolded' during visits, while others were unsure whether to follow new instructions due to a lack of explanation. This highlights a gap between clinical advice and adolescent understanding suggesting a need for more collaborative and compassionate care models.

Incorporating youth-centred frameworks into diabetes clinic operations can help address this gap. Adolescent-friendly services, as defined by the World Health Organization, should be equitable, accessible, acceptable, appropriate, and effective [27]. These principles include privacy, provider training and consistent communication, all of which were cited by participants as areas for improvement. Additionally, several adolescents expressed interest in expanding services beyond age 16 or 17, particularly in the form of diabetes camps or adult transition support, demonstrating the importance of continuity of care in reducing dropout and long-term complications.

Outside the clinic, school environments were perceived as both enabling and restrictive. While some participants benefited from understanding teachers and friends, others were excluded from physical activities or labelled as ‘different,' reflecting limited awareness among educators. This reinforces the need for school-based diabetes education stronger links between paediatric clinics and educational institutions.

### 4.4. Diabetic Health Literacy: A Holistic Perspective

The global theme of diabetic health literacy encapsulates adolescents' ability to access, understand, and apply diabetes-related knowledge across multiple domains of life. In this study, literacy was shaped not only by information delivery but also by the adolescent's emotional readiness, family dynamics, and community context. Health literacy in chronic illness is known to be a powerful predictor of adherence, glycaemic control, and long-term outcomes [28, 29].

Our findings suggest that improving diabetic health literacy in SIDS contexts like Barbados requires multi-level interventions: adolescent-friendly clinic communication, tailored psychosocial support, accessible peer networks, and a supportive school and home environment. These insights align with the broader global health imperative to deliver equitable, culturally sensitive care to youth with chronic conditions.

Our findings can also be interpreted through established theoretical frameworks. Health literacy theory, particularly Nutbeam's tripartite model of functional, interactive, and critical literacy, offers a lens to understand how adolescents acquire, interpret and apply diabetes-related knowledge [30]. Similarly, self-determination theory (SDT) highlights the importance of autonomy, competence and relatedness in sustaining motivation for chronic illness self-management [31]. These constructs align with our organising themes of autonomy, internal resilience and the role of clinic and social support. Furthermore, transition readiness frameworks, such as Got Transition's Six Core Elements and the Transition Readiness Assessment Questionnaire (TRAQ), underscore the need to prepare adolescents for independent diabetes management and seamless transfer to adult services [32, 33]. Framing our results within these models connects Caribbean experiences to international debates on adolescent chronic illness care and also aligns with the broader global health imperative to deliver equitable, culturally sensitive care to youth with chronic conditions.

Interpretation of these findings requires close attention to the sociocultural and systemic realities of Barbados and the wider Caribbean. Barbados, like many SIDS, face resource limitations in paediatric diabetes care, including limited availability of continuous glucose monitoring devices and reliance on a small multidisciplinary team concentrated at the sole institutions that can affect adolescents' capacity for consistent self-management. Family involvement remains deeply rooted in Barbadian society and while this often provides strong emotional and practical support, it can sometimes delay the development of adolescent autonomy in self-care. Similarly, cultural expectations and stigma within schools also constrain opportunities for participation and can undermine resilience [21–34]. At the institutional level, reliance on a small cadre of nurse educators, the absence of embedded psychological support, and constraints in staffing reflect systemic gaps in adolescent-focused diabetes care [35]. Recognising these influences enhances the policy relevance of our findings, underscoring the need for adolescent-friendly and resource-appropriate service models in SIDS.

## 5. Recommendations for Practice

### 5.1. Psychosocial Support

Enhance care for adolescents with chronic illnesses by integrating psychological services such as individual counselling and peer support groups to address the psychosocial challenges of adolescence including navigation of the transition process.

### 5.2. Improved Communication

Restructuring information delivery to adolescents in clinics to foster better understanding and support self-management.

### 5.3. Adolescent-Friendly Services

Ensure clinics provide a welcoming, empowering environment through staff training and services tailored to adolescents' needs, aligned with principles of accessibility, acceptability and effectiveness [36].

## 6. Limitations

Our study was the first qualitative study assessing adolescent diabetes clinic experiences in the Caribbean. We acknowledge certain limitations of this study; first, we were limited by a small sample size and exclusion of private healthcare users, and therefore, the study cannot be extrapolated to their QOL or health literacy. Second, we recognise that online interviews are not equivalent to in-person interviews; this was mitigated using video to increase participants' comfort and assess non-verbal communication. Third, access to reliable internet connection was not guaranteed and prevented two eligible participants from taking part in this study. Finally, while credibility was enhanced through reflexive practice and audit trails, member checking was not conducted, which may have limited opportunities to validate interpretations directly with participants.

## 7. Conclusions

Improving diabetic health literacy among adolescents necessitates developmentally appropriate communication, empowerment through meaningful clinic engagement and access to comprehensive psychosocial support. These elements collectively strengthen self-management capacity and enhance overall QOL—critical outcomes for adolescents living with T1DM as they navigate the transition to adulthood. To achieve sustainable improvements, diabetes specialty outpatient clinic models in SIDS such as Barbados must continue to evolve towards integrated, youth-centred approaches that address both the clinical and psychosocial dimensions of care.

## Figures and Tables

**Table 1:** Coding dictionary.

Code name 16	Abbreviation	Description—All references by participants relating to…
Duration	Dura	The length of time they have been diagnosed with diabetes
Blood sugar control	BSC	The difference in blood sugar control over time from diagnosis to the adolescent period
Diabetes management	DiabM	How they manage their blood sugars daily (inclusive of pharmacological, diet and exercise)
Hospital visits	HospV	The frequency of hospitalisations and ward reviews in adolescent period
Insulin	Insu	Their attitudes, experiences and perceptions towards insulin administration and storage
Schooling	Schol	The effect of diabetes on their school life and schooling
Relationships	Rel	How diabetes affects their relationships with family, schoolmates and friends
Feelings-chronic disease	F-CD	Their attitudes and feelings towards living with a chronic disease; in this case diabetes
Diabetes clinic	Dia-C	Their attitudes and feelings towards the diabetes clinic process
Clinic interaction	Cl-Inte	The perceived usefulness of the clinic and benefit of the physician interaction
Clinic information	Cl-Info	The perceived or experienced information provided from clinic which they use in their diabetes management
Concerns and worries	Con-W	Their concerns and worries about their chronic illness and management plan
Challenges	Chal	Their challenges and difficulties with diabetes self-management
Suggestions	Sugg	Their suggestions to improve their diabetes care and clinic visits and in turn enhance the quality of life
Quality of life	QOL	How they view their quality of life living with diabetes
Support	Sup	The support provided by relatives, schoolmates, school personnel and/or friends for medication reminders, meal preparation, doctor visits access and emotional needs

**Table 2:** Participant characteristics.

Participant no.	Duration range of disease (years)^a^	Age (years)	Sex	Paediatric diabetes clinic status^b^
P1	<5	15	F	Current
P2	5–10	16	F	Transitioning
P3	5–10	14	F	Current
P4	5–10	14	F	Current
P5	5–10	16	F	Transitioning
P6	11–15	13	M	Current
P7	5–10	13	M	Current
P8	11–15	16	F	Transitioning
P9	11–15	16	M	Transitioning
P10	<5	13	M	Current
P11	5–10	13	F	Current
P12	5–10	17	M	Transitioned

^a^Given that this was a discrete study population, duration range was used for preservation of anonymity.

^b^This refers to the participant's present status in clinic at the time of interviews. Current refers to participants enrolled in the clinic, transitioning to those participants who were of age to be transferred to adult clinic and transitioned referred to the recent transfer to adult clinic.

## Data Availability

The transcripts are not publicly available due to participant confidentiality.

## Supporting Information

Supporting Information COREQ Checklist –Exploring the Influence of a Diabetes Specialty Outpatient Clinic on Adolescents with Type 1 Diabetes in Barbados
