# Supplementary material for: Exploring the Influence of a Diabetes Specialty Outpatient Clinic on Adolescents With Type 1 Diabetes in Barbados: A Qualitative Study
Source: Pediatr Diabetes. 2025 Dec 3;2025:5454172. doi: 10.1155/pedi/5454172 (PMC12695417; doi:10.1155/pedi/5454172)
Supplement: Supporting Information — COREQ Checklist –Exploring the Influence of a Diabetes Specialty Outpatient Clinic on Adolescents with Type 1 Diabetes in Barbados [file 5454172.f1.docx]

**COREQ Checklist –"Exploring the Influence of a Diabetes Specialty Outpatient Clinic on Adolescents with Type 1 Diabetes in Barbados: A Qualitative Study"**

**Domain 1: Research Team and Reflexivity**

| **Item** | **Description** | **Response** |
| --- | --- | --- |
| 1 | **Interviewer/facilitator** | Dr. Amanda Wickham (Research Assistant) conducted all interviews. |
| 2 | **Credentials** | BMedSci, MBBS; MPH. |
| 3 | **Occupation** | Medical practitioner and research assistant. |
| 4 | **Gender** | Female. |
| 5 | **Experience and training** | Trained in qualitative interviewing and supervised by Dr. Natalie Greaves (doctoral-level qualitative researcher). |
| 6 | **Relationship established** | No prior relationship with participants. |
| 7 | **Participant knowledge of the interviewer** | Participants were informed of research purpose and process during assent. |
| 8 | **Interviewer characteristics** | PI was removed from this process to reduce interviewer bias as is a provider of care to participants. Reflexivity was addressed throughout analysis. |

**Domain 2: Study Design**

| **Item** | **Description** | **Response** |
| --- | --- | --- |
| 9 | **Methodological orientation** | Descriptive-interpretive qualitative design, thematic analysis with constant comparison. |
| 10 | **Sampling** | Purposive sampling with maximum variation aligned with eligibility criteria. |
| 11 | **Method of approach** | Anonymized clinic list and parental outreach. |
| 12 | **Sample size** | 12 adolescents. |
| 13 | **Non-participation** | 9 of 21 excluded due to migration, unreachability or refusal. |
| 14 | **Setting of data collection** | Secure online interviews via Zoom. |
| 15 | **Presence of non-participants** | Parents were asked not to be present to minimize bias. |
| 16 | **Description of sample** | Adolescents aged 13–17; duration of T1DM 3–12 years; varying clinic stages (current, transitioning, transitioned). Sex distribution included both males and females (Table 2). |
| 17 | **Interview guide** | Semi-structured interview guide (Appendix IV) developed from clinical observations; not piloted. |
| 18 | **Repeat interviews** | No repeat interviews. |
| 19 | **Audio/visual recording** | Yes; Zoom audio-visual recordings used with parental consent and child assent. |
| 20 | **Field notes** | Descriptive notes were recorded post interviews- observations of participants’ behaviors, expressions and non-verbal cues. |
| 21 | **Duration** | Interviews averaged 15 minutes. |
| 22 | **Data saturation** | Data saturation reached after the tenth interview, confirmed by stability of codes in remaining transcripts. |
| 23 | **Transcripts returned** | No transcript return to participants. |

**Domain 3: Analysis and Findings**

| **Item** | **Description** | **Response** |
| --- | --- | --- |
| 24 | **Number of data coders** | Two coders (PI + supervisor) with inter-rater checks conducted. |
| 25 | **Description of coding tree** | Yes. A 16-item coding dictionary (Table 1) was developed and described in manuscript. |
| 26 | **Derivation of themes** | Themes were derived inductively. |
| 27 | **Software** | ATLAS.ti 23 used for transcription management and coding. |
| 28 | **Participant checking** | No participant member checking. However, findings were presented to the clinical team for credibility assessment. |
| 29 | **Quotations presented** | Yes; verbatim quotations used to illustrate each theme with participant ID labels (P01-P12). |
| 30 | **Data and findings consistent** | Yes; themes aligned closely with participant narratives showing analytic consistency. |
| 31 | **Clarity of major themes** | Yes. Three organizing themes: Autonomy, Internal Resilience, and Clinic & Social Support leading to the global theme: Diabetic Health Literacy. |
| 32 | **Clarity of minor themes** | Yes; minor/embedded themes (e.g., mood, peer influence) were integrated within organizing themes. |
